# Case Report: Paroxysmal weakness of unilateral limb as an initial symptom in anti-LGI1 encephalitis: a report of five cases

**DOI:** 10.3389/fimmu.2023.1191823

**Published:** 2023-05-25

**Authors:** Shan Wang, Jirui Wang, Baizhu Li, Ning Hu, Yingbin Jin, Shiyu Han, Xiuli Shang

**Affiliations:** Department of Neurology, The First Affiliated Hospital of China Medical University, Shenyang, Liaoning, China

**Keywords:** case report, Anti-LGI1 encephalitis, autoimmune encephalitis, paroxysmal, limb weakness

## Abstract

Anti-leucine-rich glioma-inactivated 1 (LGI1) encephalitis is the second most common kind of autoimmune encephalitis following anti-N-methyl-d-aspartate receptor (NMDAR) encephalitis. Anti-LGI1 encephalitis is characterized by cognitive impairment or rapid progressive dementia, psychiatric disorders, epileptic seizures, faciobrachial dystonic seizures (FBDS), and refractory hyponatremia. Recently, we found an atypical manifestation of anti-LGI1 encephalitis, in which paroxysmal limb weakness was the initial symptom. In this report, we describe five cases of anti-LGI1 encephalitis with paroxysmal limb weakness. Patients had similar presentations, where a sudden weakness involving a unilateral limb was observed, which lasted several seconds and occurred dozens of times each day, with the anti-LGI1 antibody being positive in both serum and cerebrospinal fluid (CSF). FBDS occurred after a mean of 12 days following paroxysmal limb weakness in three of five patients (Cases 1, 4, and 5). All patients were given high-dose steroid therapy, which had a good effect on their condition. Based on this report, we suggest that paroxysmal unilateral weakness may be a kind of epilepsy and be connected to FBDS. As an unusual neurological presentation, paroxysmal weakness can be included in the clinical manifestations of anti-LGI1 encephalitis, helping to raise awareness of the recognition of anti-LGI1 encephalitis in patients with this symptom and leading to early diagnosis and early treatment, which would contribute to improved clinical outcomes.

## Introduction

First proposed in 2010 (1), anti-leucine-rich glioma-inactivated 1 (LGI1) encephalitis is the second most common type of autoimmune encephalitis (2), accounting for about 12.8% in China (3). It is characterized by rapid progressive dementia, psychiatric disorders, epileptic seizures, and faciobrachial dystonic seizures (FBDS) (4).

At the beginning of the disease, some prodromal symptoms can be found in patients with anti-LGI1 encephalitis, such as paroxysmal dizziness spells, seizures, fatigue, or drowsiness (5, 6). Qiao reported a patient who suffered paroxysmal hyperhidrosis as an initial symptom (7). These non-specific or uncommon manifestations prevent patients from early diagnosis and early treatment. The paroxysmal weakness of the unilateral limb reported in our patients has never been seen in anti-LGI1 encephalitis, and this is the only case report documented on record.

Herein, we describe five cases of anti-LGI1 encephalitis with paroxysmal limb weakness as an initial symptom.

## Patients and methods

A total of five patients with anti-LGI1 encephalitis, treated at the First Affiliated Hospital of China Medical University (Shenyang, China) between 2019 and 2022 (58 cases of LGI-1 encephalitis were treated in total during the same period) were investigated. This is a retrospective study of the patient characteristics and treatment outcomes, and ethical committee approval was not required for this study, but written informed consent of all patients was obtained. The clinical features of the five cases are included in **Table 1**. In all five patients, the diagnosis of anti-LGI1 encephalitis was not only dependent on their clinical characteristics and magnetic resonance imaging (MRI) of brain, but also positive anti-LGI1 antibodies in serum and CSF (8).

**Table 1:** Patient’s clinical features.

	Case 1	Case 2	Case 3	Case 4	Case 5
Age (years), sex	60, male	33, female	61, male	59. male	55, male
Involved limb	Left arm and leg	Left leg	Right arm	Left arm	Left arm
FBDS	Yes	No	No	Yes	Yes
Days from weakness to FBDS onset	20	–	–	13	5
Short-term memory deterioration	Yes	No	Yes	Yes	No
Psychiatric disorder	No	No	Yes	Yes	No
EEG	Normal	Normal	Normal	Normal	Normal
Cranial MRI	T2-weighted and FLAIR hyper- intensity in the right hippocampus	Normal	T2-weighted and FLAIR hyper-intensity in the left hippocampus	Normal	Normal
CSF analysis	Normal	Normal	Normal	Normal	Normal
Anti-LGI1 positivity and titer	Serum 1:100 CSF 1:3.2	Serum 1:30 CSF1:10	Serum 1:30 CSF1:30	Serum 1:30 CSF1:10	Serum 1:100 CSF1:10
Immunotherapy	Methylprednisolone 500mg for 5 days, half decrement every 3 days, subsequent oral steroid tapering	Methylprednisolone 1000mg for 3 days, half decrement every 3 days, subsequent oral steroid tapering	Methylprednisolone 500mg for 5 days, half decrement every 3 days, subsequent oral steroid tapering	Methylprednisolone 500mg for 5 days, half decrement every 3 days, subsequent oral steroid tapering	Methylprednisolone 500mg for 7 days, half decrement every 3 days, subsequent oral steroid tapering
Outcome (follow-up)	Occasional paroxysmal limb weakness and FBDS (3 months). recurrence (20 months), FBDS reduced after methylprednisolone 500mg, weakness and FBDS disappeared (28 months)	Paroxysmal limb weakness disappeared (3 months)	Paroxysmal limb weakness disappeared (2 months) Short-term memory deterioration (4 months)	Occasional paroxysmal limb weakness and FBDS, normal mental status (2 months)	Paroxysmal limb weakness and FBDS disappeared, normal mental status (3 months)

The anti-LGI1 antibody was detected by cell based assay (CBA) and tissue based assay (TBA), where the principle was to transfect the gene of the anti-LGI1 encephalitis antigen into mammalian cells by plasmid pcDNA3.1. The corresponding antigen was specifically expressed in mammalian cells, and green fluorescent protein was expressed during transfection as an internal reference for detection. Then, the transfected cells were fixed on a 96-well microplate, and the semi-quantitative detection of specific antibodies in human serum and cerebrospinal fluid samples was carried out using the principle of indirect immunofluorescence. Then, observed under a fluorescence microscope for result judgment, the cells were first observed through the green channel to check for successful transfection. If the plasmid transfection was successful, green fluorescence could be observed in the cells. Then, the cells were observed through the red channel, and if there was a clear red fluorescence in the transfected cell membrane of the sample well, it was considered a positive sample. If there was no clear red fluorescence in the transfected cell membrane of the sample well or if untransfected cells also showed red fluorescence, it was considered a negative sample. The results could be further confirmed by overlapping the green and red channels. Samples with positive results were selected 3 to 5 fields of view under a microscope, comparing the red fluorescence with the control sample. The positive titer value was given by comparing the intensity of the red fluorescence with that of the control sample.

Different sample types had different starting dilutions for the CBA method. The starting dilution for serum samples was 1:10, while the starting dilution for cerebrospinal fluid samples was 1:1. Usually, when the titer of anti-LGI1 encephalitis antibody reaches 1:10 in serum and 1:1 in cerebrospinal fluid at the same time, a positive diagnosis can be made.

## Result

### Case 1

**Figure 1:**
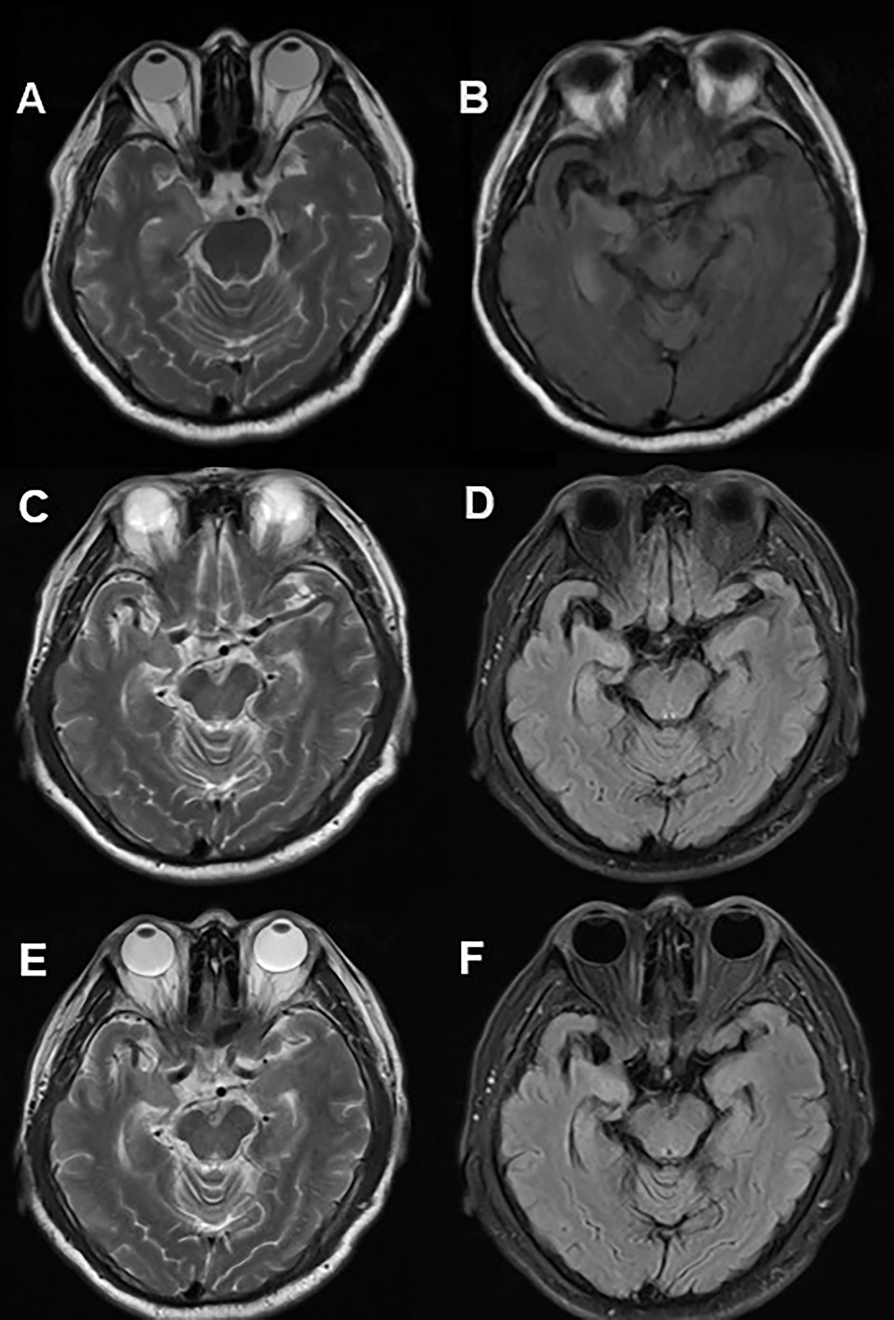
Cranial MRI in Case 1. Axial T2 weighted **(A)** and FLAIR **(B)** sequences showed hyper-intensity in the right hippocampus. 20 months after immunotherapy, a control cranial MRI showed a significant reduction of right hippocampus hyper-intensity in T2 **(C)** weighted and FLAIR **(D)** sequences. 28 months after immunotherapy, no significant changes contrasted **(E, F)** with **(C, D)**.

A 60-year-old man was admitted to hospital due to paroxysmal weakness in his left limbs. At first, these episodes occurred once every few days and lasted for several seconds. However, over time, the frequency of weakness increased to 3-4 times an hour, and he also experienced a sensation of ants crawling on his left arm and leg. About 20 days after the onset of weakness, he started experiencing involuntary movements in his left limbs and face, which lasted for several seconds and occurred multiple times a day. These events were classified as FBDS. A cranial MRI showed hyper-intensity in the right hippocampus on T2 and fluid attenuated inversion recovery (FLAIR) sequences (**Figure 1**). Lab tests revealed the positivity of anti-LGI1 antibody in both serum (1:100) and CSF (1:3.2). The patient was diagnosed with anti-LGI1 encephalitis and was treated with methylprednisolone at 500mg for 5 days, with a gradual tapering every 3 days, followed by oral prednisone. During his hospitalization, the frequency of the repetitive weakness and FBDS reduced to once every few days. Three months after the initial testing, anti-LGI1 antibody in serum was negative; however it increased to 1:1000 in the 20th month after disease onset. At this point, the patient again developed paroxysmal weakness in his left limbs and FBDS, which occurred multiple times a day, and was readmitted to the neurological department. However, a follow-up cranial MRI showed a significant reduction of right hippocampus hyper-intensity on T2 weighted and FLAIR sequences (**Figure 1**). Another round of corticosteroid impulse therapy was also effective, and the symptoms was progressively improved. Over the course of the 28-month follow-up period, paroxysmal left limb weakness and FBDS disappeared, and anti-LGI1 antibody in serum was negative.

### Case 2

A 33-year-old woman complained of paroxysmal weakness in her left leg. She first noticed the symptoms when she wanted to rise from a chair but failed because of the powerless state of her left leg, which returned to normal after 5 seconds. Then she found the episodes of repetitive weakness occurred more than 10 times each day without any apparent triggers. She couldn’t walk during the onset of weakness and sometimes even fell down. Cranial MRI and EEGs did not reveal any abnormalities. Low-dose carbamazepine was administered as an experimental treatment but did nothing to relieve the weakness. Lab tests revealed the positivity of anti-LGI1 antibody in both serum (1:30) and CSF (1:10). Based on this information, the patient was diagnosed with anti-LGI1 encephalitis and treated with methylprednisolone of 1000mg for 3 days, with a decrement by half every 3 days, By the 4th day of immunotherapy, the weakness had disappeared and only reoccurred seven times during her hospitalization. After discharge from hospital, she continued taking prednisone orally. During the 3 months of follow-up, she did not experience any recurrence of the weakness (**Supplementary Video 1**).

### Case 3

**Figure 2:**
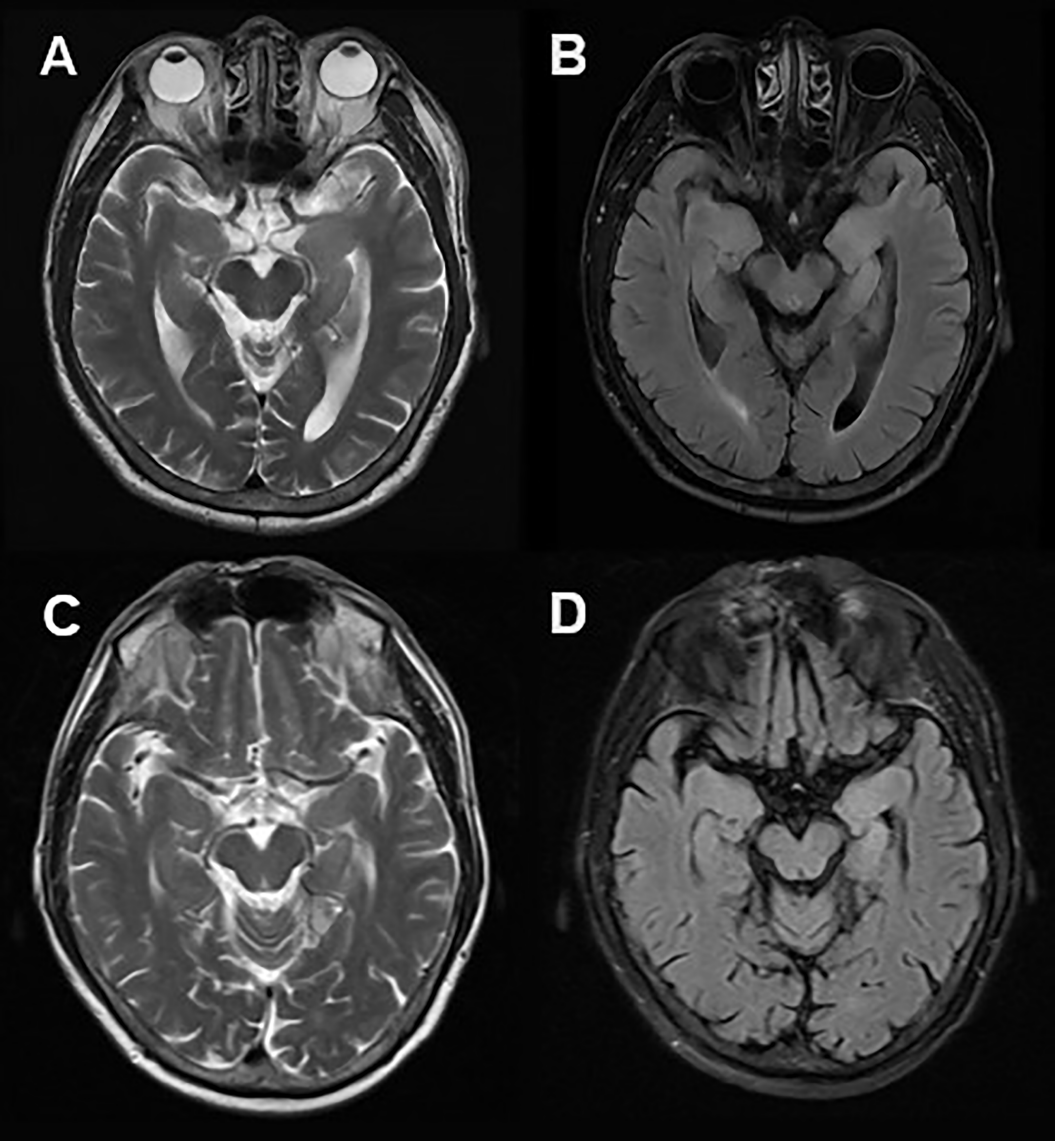
Cranial MRI in Case 3. Axial T2 weighted **(A)** and FLAIR **(B)** sequences showed hyper-intensity in the left hippocampus. 2 months after immunotherapy, a control cranial MRI showed a significant reduction of right hippocampus hyper-intensity in T2 weighted **(C)** and FLAIR **(D)** sequences.

A 61-year-old man was admitted to hospital because of a 2-month history of repetitive weakness with short-term memory deterioration and psychiatric disorders. During an episode, he would yell at people and seemed to be out of control, and it was noticed that he couldn’t raise his right arm. Additionally, he displayed irritability and visual hallucinations, such as watching somebody else taking a tumble. These symptoms lasted for about 2 minutes each time and occurred more than 10 times a day. The patient also had impaired memory, as evidenced by forgetting what he ate for breakfast, and he scored 20/30 on the Montreal Cognitive Assessment (MoCA) and 16/30 on the Mini-Mental State Examination (MMSE). Cranial MRI showed hyper-intensity on T2 and FLAIR sequence in the left hippocampus (**Figure 2**). Lab tests revealed the presence of anti-LGI1 antibody in both serum (1:30) and CSF (1:30). Based on these findings, he was diagnosed with anti-LGI1 encephalitis and was treated with methylprednisolone of 500mg for 5 days, with a decrement by half every 3 days, followed by oral prednisone. During his treatment, his repetitive weakness disappeared, and his memory improved (with a score of 21/30 on MoCA and 26/30 on MMSE). After 4 months of follow-up, the patient had no recurrence of his symptoms.

### Case 4

A 59-year-old man was admitted to hospital because of repetitive weakness and numbness in his left arm, which was initially diagnosed as cerebral infarction. However, despite treatment, his condition did not improve and instead worsened over time. The frequency of his weakness increased from less than 10 times a day to dozens of times each day. He also developed dementia and exhibited abnormal behavior, which made it difficult for him to communicate with people around him. About 13 days after weakness onset, he began experiencing paroxysmal convulsions in his left arm, leg, and face. These convulsions, known as FBDS, lasted for 2 seconds and occurred 10 times every hour, leading to him falling. However, his cranial MRI and EEGs revealed no abnormalities. However, lab tests revealed the presence of anti-LGI1 antibody in both serum (1:30) and CSF (1:10). Based on these findings, he was diagnosed with anti-LGI1 encephalitis and was treated with methylprednisolone of 500mg for 5 days, with a half-dose reduction every 3 days, followed by oral prednisone. Before being discharged from hospital, the frequency of FBDS decreased sharply to about 20 times a day, and his overall mental state improved, although he still had some memory impairment. Over the following 3 months of follow-up, he recovered, and paroxysmal weakness and FBDS occurred occasionally.

### Case 5

A 55-year-old male complained of paroxysmal weakness in his left arm, manifested in him not being able to fully clench his fist for 10 seconds, and occurred 2 or 3 times daily. Five days later, he suffered paroxysmal convulsions affecting his left arm and face, accompanied by more frequent episodes of weakness occurring more than 10 times per day. Despite undergoing cranial MRI and EEGs tests, no abnormalities were found. However, lab tests revealed the presence of anti-LGI1 antibody in both serum (1:100) and CSF (1:10). He was diagnosed with anti-LGI1 encephalitis and treated with methylprednisolone of 500mg for 7 days, with a half-dose reduction every 3 days, followed by oral prednisone. At the 7th day of treatment, repetitive weakness and FBDS had disappeared. During the 3 months of follow-up, he did not experience any recurrence of symptoms (**Supplementary Video 2**).

### Treatment and outcome

All five patients received high doses of steroid therapy at an initial dose of 500 or 1000 mg a day. Their conditions rapidly improved, with the frequency of paroxysmal weakness and FBDS decreasing, and their mental states became better.

At the last follow-up, there were no relapses in four patients (Cases 2–5). Case 1 suffered a relapse in the 20th month, and a new round of corticosteroid impulse therapy was still effective.

Some studies suggest that when the antibody titer in the cerebrospinal fluid is less than 1:10, it may be falsely positive and anti-LGI1 encephalitis cannot be diagnosed. At this time, a patient’s clinical symptoms and imaging results should be considered to diagnosis encephalitis. In these cases, although there were low titers of antibodies in the cerebrospinal fluid, the diagnosis of encephalitis was confirmed based on the serum antibody titer, as well as the patient’s clinical symptoms and imaging results, and the effectiveness of immunotherapy.

## Discussion

This report describes five patients with anti-LGI1 encephalitis who had paroxysmal unilateral weakness as an initial symptom, accompanied by other clinical manifestations, such as FBDS, memory deficits, and psychiatric disorder.

As the most common limbic encephalitis, anti-LGI1 encephalitis is an acute or subacute disorder, and mainly affects adults between the ages of 30 to 80 with a higher incidence in men (9). In our report, 4(80%) of five patients were men, and the average age was 53.6 years (median, 59 years; range, 33–61 years), which agreed with the results of previous studies. LGI1, a secreted neuronal protein, forms a trans-synaptic complex with the presynaptic disintegrin and metalloproteinase domain-containing protein 22 (ADAM22), which interacts with AMPA receptors, and the postsynaptic ADAM23, which interacts with voltage-gated potassium channels Kv1.1 (10, 11). In addition, IgG secreted in patients with anti-LGI1 encephalitis disrupts the LGI1 signal of presynaptic and postsynaptic neurons, causing neuronal hyperexcitability and reversible memory deficits (12).

FBDS are characteristic symptoms of anti-LGI1 encephalitis and are considered pathognomonic, with brief inflexible posturing events typically lasting several seconds, usually less than 3 seconds, and occurring dozens of times during the day. FBDS involve the face and the ipsilateral arm, and sometimes the ipsilateral leg. The origin of FBDS is still a topic of heated discussion with no definitive conclusions. Because of epileptiform EEG changes, some researchers considered that FBDS are atypical epileptic phenomena (13), and that FBDS arises from network dysfunction between cortical and subcortical regions. Imaging abnormalities in basal ganglia have also been discovered in patients who experience FBDS (14, 15). Immunotherapy has been found to be effective in treating FBDS and can lead to their cessation (6, 16).

This abnormal pattern of weakness in the patients with anti-LGI1 encephalitis lasted for several seconds every time and occurred hundreds of times per day. This weakness involved a unilateral limb without any apparent inducement and did not respond to antiepileptic drugs. However, it showed a favorable response to high-dose steroid therapy. Following with the weakness, involuntary movements of the affected limbs and ipsilateral face, which are clinically recognized as FBDS, appeared gradually at the same time in three (Cases 1, 4, and 5) of the five cases. The mean duration from weakness to FBDS onset was about 12 days.

Paroxysmal limb weakness can be seen in patients with paroxysmal kinesigenic dyskinesia (PKD), but this type of weakness typically occurs after sudden motion state changes and usually manifests in the teen years. Furthermore, the relief of weakness in PKD patients is associated with immunotherapy but not carbamazepine (17). Therefore, in our patients, the weakness was considered a symptom of encephalitis.

The clinical manifestations of paroxysmal limb weakness in the cases we reported are similar to FBDS. In addition, immunotherapy has been found to be highly effective in preventing weakness and FBDS. Therefore, considering the temporal relationship between paroxysmal limb weakness and FBDS, we hypothesize that weakness may be a prodromal manifestation of FBDS and the mechanism of the paroxysmal limb weakness is similar to that of FBDS, suggesting that it is a form of epilepsy. However, we were unable to confirm this hypothesis, as we did not find any abnormalities in EEGs. Additionally, due to timely treatment or short-term follow-up, we did not find the appearance of FBDS in Case 2 and Case 3. As our report only analyzed five cases, it remains unclear whether the relationship between paroxysmal limb weakness and FBDS is coincidental or causal. To our knowledge, paroxysmal limb weakness has not been previously reported as an initial symptom of anti-LGI1 encephalitis or associated with FBDS.

Various paroxysmal and repetitive manifestations have been reported in the literature. Paroxysmal dizziness spells (PDS) were first proposed by Gadoth; these are partial seizures or aura phenomena which cannot be detected by EEGs (6). Another study by Beimer et al. identified a patient with anti-LGI1 encephalitis who suffered from ictal speech and manual automatisms, and video EEGs confirmed that the abnormal symptoms were a special seizure type (18). Qiao et al. also suggested that paroxysmal hyperhidrosis could be a possible form of epilepsy, which is different from an autonomic symptom (6). Furthermore, video EEGs observed epileptic waves during recurrent chest discomfort for the patient reported by Lee (19). These paroxysmal or ictal symptoms could be categorized as a specific type of seizure.

This article reports on the characteristics of paroxysmal limb weakness and analyzed its features, which are easily misdiagnosed as TIA or epilepsy in clinical practice. Finally, it was confirmed as a diagnosis of anti-LGI1 encephalitis. This recurrent type of symptom has not been reported internationally. Due to the limitations of sample size and time, this article cannot yet determine the overall incidence and long-term prognosis of this paroxysmal symptom and requires further observation with larger sample sizes.

While the association of weakness with FBDS remains unclear, paroxysmal unilateral limb weakness has been identified as an initial symptom of anti-LGI1 encephalitis. When paroxysmal unilateral symptoms, such as weakness of limbs, convulsions, and hyperhidrosis, are noticed, it is essential for patients to undergo LGI1 antibody testing for accurate diagnosis and treatment of this unusual disease.

Based on this report, we demonstrate an unusual neurological presentation that is included in the clinical manifestations of anti-LGI1 encephalitis, helping to recognize anti-LGI1 encephalitis in patients with this symptom and leading to early diagnosis and early treatment, which would contribute to improved clinical outcomes.

## Data availability statement

The original contributions presented in the study are included in the article/**Supplementary Material**. Further inquiries can be directed to the corresponding author.

## Ethics statement

Ethical review and approval was not required for the study on human participants in accordance with the local legislation and institutional requirements. The patients/participants provided their written informed consent to participate in this study. Written informed consent was obtained from the individual(s) for the publication of any potentially identifiable images or data included in this article.

## Author contributions

SW wrote the manuscript; SW and XS revised the manuscript; all authors contributed to follow-up, information collection, and approved the submitted version.

## Supplementary material

Supplementary Video 1 The patient of case 2 couldn't move with her powerless left leg.

Supplementary Video 2 The patient of case 5 couldn't fully clench his fist with his powerless left arm.
